# Food Consumption Patterns among U.S. Children from Birth to 23 Months of Age, 2009–2014

**DOI:** 10.3390/nu9090942

**Published:** 2017-08-26

**Authors:** Heather C. Hamner, Cria G. Perrine, Priya M. Gupta, Kirsten A. Herrick, Mary E. Cogswell

**Affiliations:** 1 National Center for Chronic Disease Prevention and Health Promotion, Centers for Disease Control and Prevention (CDC), Atlanta, GA 30341, USA; hgk3@cdc.gov (C.G.P.); kso7@cdc.gov (P.M.G.); mec0@cdc.gov (M.E.C.); 2 National Center for Health Statistics, Centers for Disease Control and Prevention (CDC), Hyattsville, MD 20782, USA; goi7@cdc.gov

**Keywords:** young children, food consumption patterns, NHANES, sugar-sweetened beverages, fruit and vegetables, race/Hispanic origin

## Abstract

Early dietary patterns can have long-term health consequences. This study describes food consumption patterns among US children ≤23 months. We used one 24 h dietary recall from the National Health and Nutrition Examination Survey 2009–2014 to estimate the percentage of children ≤23 months who consumed selected food/beverage categories on any given day by age and race/Hispanic origin. Among 0 to 5 month olds, 42.9% (95% Confidence Interval (CI): 37.0%, 49.1%) consumed breast milk, with non-Hispanic blacks less likely (21.2%, 95% CI: 13.2%, 32.2%) compared with non-Hispanic whites (49.0%, 95% CI: 39.0%, 59.1%) (*p* < 0.001). The percentage of children consuming vegetables was 57.4%, 48.2%, and 45.1% for ages 6 to 11, 12 to 18 and 19 to 23 months, respectively (*p* < 0.01 for trend). The percentage of children consuming sugar-sweetened beverages was 6.6%, 31.8% and 38.3% for ages 6 to 11, 12 to 18 and 19 to 23 months, respectively (*p* < 0.01 for trend). Among children aged ≥6 months, lower percentages of non-Hispanic black and Hispanic children consumed vegetables, and higher percentages consumed sugar-sweetened beverages and 100% juice compared with non-Hispanic white children, although differences were not always statistically significant. Compared with children in the second year of life, a higher percentage of children 6 to 11 months of age consumed vegetables and a lower percentage consumed 100% juice, sugar-sweetened beverages, snacks, or sweets; with differences by race/Hispanic origin. These data may be relevant to the upcoming 2020–2025 federal dietary guidelines.

## 1. Introduction

The first two years of a child’s life are an important time period for introducing and establishing healthy eating patterns through encouraging selected foods/beverage types such as breast milk, fruits, and vegetables that provide a variety of flavors; while limiting the consumption of foods that may contribute to consumption of high calories and low nutrient density foods/beverages [1]. For example, research has indicated that children who were breastfed were more likely to have a number of healthier dietary behaviors at six years of age [2]. Similarly, children who consumed sugar-sweetened beverages during infancy were more likely to consume a sugar-sweetened beverage at six years of age [3]. Currently, the U.S. Dietary Guidelines for Americans 2015–2020 are intended for the U.S. population from ages two and older, and provide guidance on healthy dietary eating patterns [4]. There are no U.S. federal dietary guidelines in existence for children younger than two years of age; however, federal dietary guidelines for this age group are expected to be incorporated, or released in conjunction with, the upcoming 2020–2025 Dietary Guidelines for Americans [5]. Until then, recommendations on infant and toddler feeding are limited to publications or reports from organizations such as The American Academy of Pediatrics [6,7].

Studies assessing infant and toddler feeding patterns are dated [8,9]. The objective of this study is to describe the consumption of selected food and beverage categories among a nationally representative sample of U.S. infant and toddlers from birth through 23 months of age, by age and race/Hispanic origin. These data provide an assessment of current selected food and beverage consumption among U.S. infants and toddlers, and may provide baseline data that could be relevant to the upcoming Dietary Guidelines for this age group [5].

## 2. Materials and Methods

### 2.1. National Health and Nutrition Examination Survey

The National Health and Nutrition Examination Survey (NHANES) is an ongoing, nationally representative survey of the non-institutionalized civilian US population, and uses a stratified multistage probability design [10]. Detailed information on the study designs and methods are available elsewhere [11,12]. Briefly, the survey includes a household interview and a physical examination conducted in a Mobile Examination Center (MEC). All participants provide written informed consent. A parent or guardian provides written consent for minors to participate. Data are released in two-year cycles, and this analysis combines data from 2009–2010, 2011–2012, and 2013–2014. The NHANES protocol was approved by the National Center of Health Statistics Research Ethics Review Board. Response rates for children aged 1–5 years who underwent the physical examination were 86.8% in 2009–2010, 77.6% in 2011–2012, and 74.6% in 2013–2014 [13].

### 2.2. Dietary Intake and Food Group Categorization

Two 24-hour dietary recalls are collected as part of NHANES; the initial recall is collected in-person in the MEC, and the second, via telephone, 3–10 days later. For this analysis, the initial dietary recall was used and data are representative of a “given day’s” intake for the population. Dietary information is collected by trained interviewers using the Automated Multiple Pass Method (AMPM) [14], a computer-assisted program designed to standardize data collection and increase the probability of complete reporting of all foods and beverages, including breast milk, consumed in the previous 24 h. A proxy (generally a parent) who is most familiar with the child’s intake, completes the dietary recall.

Food groups were identified using the United States Department of Agriculture’s (USDA) What We Eat in America (WWEIA) categories [15] that describe foods as consumed (i.e., Pizza, Sandwiches), and not individual components of foods. WWEIA categories are comprised of mutually exclusive large food categories which are further divided into smaller subcategories. The subcategories and the associated WWEIA food code were used to develop the 15 food and beverage categories that are reported for the current study (Breast milk; Formula; Reduced or low or nonfat milk; Flavored milk and/or milk substitute; Water; 100% Juice; Sugar-sweetened beverages; Fruit; Vegetables, excluding white potatoes; Protein; Grains; Mixed Dishes; Snacks; and Sweets) (Supplemental Table S1). Two additional food categories (White potatoes and Baby foods) are presented in the Supplemental Table (Table S2), and their associated WWEIA food codes are provided in Table S1. The WWEIA categories are updated with new foods codes for every NHANES dietary data release [16,17,18].

### 2.3. Analytic Sample

We limited our analyses to children who were ≤23 months of age at the time of the physical examination (*n* = 1912). Children were excluded if the dietary interview was not completed (*n* = 62) or if the dietary intake record was coded as not reliable (*n* = 26). This left a final sample size of 1824 children ≤23 months of age.

### 2.4. Covariates

We described the analytic samples by the child’s age (birth to five months, six to 11 months, 12 to 18 months, and 19 to 23 months), and the child’s race/Hispanic origin (non-Hispanic white, non-Hispanic black, and Hispanic). Race/Hispanic origin was based on parental response. Children who were identified as other race, including multi-racial, were included in analyses but were not presented as a separate group.

### 2.5. Statistical Analyses

Using data from the first 24-hour dietary recall, we estimated the percentage of children who reported consuming (hereafter referred to as “consumed”) any amount of a food or beverage in predefined food or beverage categories in the previous 24 h. High or low consumption was not determined; rather the frequency of any consumption versus no consumption on any given day was determined for the specified population group. Estimates and 95% confidence intervals (CI) (using the Wald method) were calculated for all, by age group, and by race/Hispanic origin. We used Chi-square tests, *t*-tests (a priori pairwise comparisons were done by age and within each age group by race/Hispanic origin), and orthogonal linear tests for trend by age (6–23 months only) to determine whether food consumption frequencies differed by age.

SPSS Complex Samples Design version 23.0 (SPSS Inc., Chicago, IL, USA) was used in all analyses to account for the complex survey design. Analyses were weighted using day one dietary weights which adjust for over sampling, non-response, non-coverage, and day of the week [19,20].

## 3. Results

Demographic characteristics of U.S. children birth to 23 months of age in 2009–2014 are shown in Table 1. Almost a quarter (23.7%) of children were zero to five months, 27.2% were six to 11 months, 30.4% were 12 to 18 months, and 18.7% were 19 to 23 months old. About half (50.7%) of children were non-Hispanic white, 13.2% were non-Hispanic black, and 27.5% were Hispanic.

Table 2 shows the percentage of U.S. children who consumed select food or beverage categories by age. On any given day, among children birth to five months of age, 42.9% consumed breast milk, 70.5% formula, 11.4% water, 2.1% 100% juice, 1.7% sugar-sweetened beverages, 8.2% fruit, 8.1% vegetables, 0.8% protein, and 21.1% grains.

Among children 6 to 23 months, the percentage who consumed beverages or other liquids varied across age. Several examples are presented. The percentage of children aged 6 to 23 months who consumed breast milk or formula on any given day decreased significantly by age (six to 11 months: 25.8%, 79.3%; 12 to 18 months: 7.6%, 9.4%; and 19 to 23 months: 5.5%, 5.9%, respectively, *p* < 0.001 for trend within beverage category). Compared with children aged from six to 11 months, a higher percentage of children aged from 12 to 18 and 19 to 23 months consumed water, flavored milk and/or milk substitutes, 100% juice, or sugar-sweetened beverages on any given day. For example, a higher percentage of children aged 12 to 18 and 19 to 23 months consumed 100% juice or sugar-sweetened beverages on any given day compared with children aged six to 11 months (six to 11 months: 12.3%, 6.6%; 12 to 18 months: 49.1%, 31.8%; and 19 to 23 months: 51.1%, 38.3%, respectively, *p <* 0.001 for each *t*-test within beverage category). In the second year of life, the percentage of children who consumed whole milk or reduced or low or nonfat milk on any given day differed between 12 to 18 month olds and 19 to 23 month olds (whole milk: 70.0%, 55.3%; reduced or low or nonfat milk: 17.3%, 30.8%; respectively, *p* < 0.05 for each *t*-test within beverage category).

The percentage of children aged six to 23 months who reported consuming fruit on any given day did not vary significantly by age; in contrast to significant differences observed for other food categories. Select examples of differences are presented. On any given day, the percentage of children who consumed vegetables was 57.4% among six to 11 month-olds which was higher than both 12 to 18 month olds (48.2%) and 19 to 23 month olds (45.1%) (*p <* 0.05 for each *t*-test). In contrast, compared with children aged six to 11 months, a higher percentage of children aged 12 to 18 and 19 to 23 months consumed protein, grains, mixed dishes, snacks, and sweets. Another example, 13.6% of children aged six to 11 months consumed sweets compared with 58.9% of 12 to 18 months olds and 62.9% of 19 to 23 months olds (*p* < 0.05, for each *t*-test) on any given day.

The percentage of U.S. children who consumed select food or beverage categories by age and race/Hispanic origin are shown in Table 3. Select examples of differences are presented. Among children aged zero to five months, a higher percentage of non-Hispanic white and Hispanic children consumed breast milk than non-Hispanic black children on any given day (49.0%, 41.9%, 21.2%, respectively, *p* < 0.001, for each *t*-test). Among children aged six to 11 months, a higher percentage of Hispanic children (18.2%) consumed 100% juice compared with non-Hispanic white children (8.6%) (*p* < 0.05, *t*-test) on any given day. Among children aged ≥12 months, non-Hispanic white children were less likely to consume 100% juice or sugar-sweetened beverages than non-Hispanic black children on any given day (*p* < 0.001, for each *t*-test).

Among children aged zero to five months, a higher percentage of non-Hispanic black children consumed grains on any given day than non-Hispanic white or Hispanic children (34.2%, 20.8%, 16.7%, respectively, *p* < 0.05, for each *t*-test). Among children six to 11 months, a higher percentage of non-Hispanic white children consumed fruit than non-Hispanic black or Hispanic children (75.4%, 49.4%, 64.0%, respectively, *p* < 0.05, for each *t*-test) on any given day. Among children aged 12 to 18 months, a higher percentage of non-Hispanic white children consumed vegetables than non-Hispanic black or Hispanic children (57.1%, 41.2%, 36.8%, respectively, *p* < 0.05, for each *t*-test) on any given day. Lastly, among children 19 to 23 months, a lower percentage of Hispanic children consumed snacks than non-Hispanic black or non-Hispanic white children (47.0%, 70.4%, 61.7%, respectively, *p* < 0.05, for each *t*-test) on any given day.

## 4. Discussion

On any given day, U.S. children ≤23 months consumed selected food and beverage categories such as breast milk, formula, milk, water, fruit, vegetables, grains, and proteins; however, the percentage of children who consumed these different categories varied by age and by race/Hispanic origin. For example, an overall proportion of four in ten children from zero to five months old consumed breast milk, but a lower percentage of non-Hispanic black children from zero to five months old consumed breast milk compared with non-Hispanic white and Hispanic children on any given day. Another example, compared with children in the second year of life, a higher percentage of children from six to 11 months of age consumed vegetables and a lower percentage consumed 100% juice, sugar-sweetened beverages, snacks, or sweets; with differences by race/Hispanic origin. We discuss our results as compared with other studies and highlight several differences by race/Hispanic origin.

The 2008 Feeding Infants and Toddlers Study (FITS) is the most recent national sample of children ≤47 months that assessed dietary intake of children [21]. FITS 2008 was a cross-sectional survey in which households from across the U.S. were sampled from a consumer database; the data were weighted to account for the nonresponse and underrepresentation of some subgroups of the population [21]. When comparing FITS 2008 to NHANES 2009–2014 data, there are examples of similar estimates of food and beverage consumption.

Siega-Riz et al. report that among children in FITS 2008, 42.2% of infants from four to 5.9 months of age consumed breast milk, and 65.3% consumed formula [8]. This is comparable to estimates we found in NHANES 2009–2014: 42.9% consumed breast milk and 70.5% consumed formula, respectively, among infants aged zero to five months. Compared with children aged six to 11 months old, we observed a higher percentage of children in the second year of life, on any given day, who consumed 100% juice, sugar-sweetened beverages, snacks, and sweets and a lower percentage who consumed vegetables. FITS 2008 data indicate a similar pattern; a higher percentage of children in older age groups (i.e., 12 to 23.9 months) consumed 100% juice, sweetened beverages, salty snacks, and desserts and candy as compared with children aged six to 11.9 months [8].

Not all observations in FITS 2008 and NHANES 2009–2014 are similar. A lower percentage of children in FITS 2008 consumed salty snacks (six to 8.9 months: 0.1%; nine to 11.9 months: 2.3%; 12 to 14.9 months: 10.4%; 21 to 23.9 months: 23.7%) compared with NHANES 2009–2014 on any snack consumption (six to 11 months: 12.5%, 12 to 18 months: 57.6%, 19 to 23 months: 56.7%) or NHANES 2009–2014 savory snack consumption (six to 11 months: 5.6%, 12 to 18 months: 25.1%, 19 to 23 months: 34.9%) [8]. These differences may be due to categorization of any snacks, savory snacks, or salty snacks in the two surveys.

In the current analysis of NHANES 2009–2014, we found differences in the percentage of children who consumed selected food and beverage categories by race/Hispanic origin within each age group. Among children 0 to 5 months of age, Non-Hispanic black children were less likely to have consumed breast milk in the previous 24 h than non-Hispanic white or Hispanic children (21.2%, 49.0%, 41.9%, respectively). The U.S. National Immunization Survey (NIS) assesses national and state breastfeeding rates in the U.S. [22]. Breastfeeding estimates between NIS and NHANES 2009–2014 are not comparable because of differences in methodology; NIS data provide estimates of breastfeeding rates in the U.S.; whereas NHANES can provide an estimate of the percentage of children who drank breast milk on a given day. However, both NIS and NHANES 2009–2014 indicate a lower percentage of breastfeeding among non-Hispanic black women compared with non-Hispanic white and Hispanic women [22].

Data from FITS 2008 have not assessed differences by race/Hispanic origin. Earlier data from FITS 2002 reported differences of food and beverage consumption or meal patterns between Hispanic and non-Hispanic children [23,24]; however, these data are 15 years old, making comparisons difficult. The differences in race/Hispanic origin we report using NHANES 2009–2014 data may be related to a number of factors including home food availability, parental diet, and familial eating habits [25,26], or access to healthy food options [27].

Differences between FITS 2008 and NHANES 2009–2014 may be due to study design, the population surveyed, food or beverage categorization, a change in eating patterns over time, or some other factor [21]. NHANES data allow for nationally representative race/Hispanic origin specific estimates of the percentage of U.S. children ≤23 months who have consumed various food and beverage categories, which was not feasible with FITS 2008 given its study design, and provide an update to the FITS 2008 data.

This study had several limitations. We examined intake on a given day; therefore, we may have underestimated the consumption of foods that are not consumed on a daily basis. We did not assess the amount of food consumed, only that it was consumed. Thus for example, although a higher percentage of children aged six to 11 months of age may consume vegetables, they may consume a smaller amount at each serving than older children, due to lower energy needs. However, early dietary patterns, including repeated exposure to foods like fruits and vegetables, have been associated with an increased acceptance of these fruits and vegetables [28]; therefore, any consumption of foods/beverages is still informative. A future analysis could examine amounts of specific foods consumed, but was beyond the scope of this paper. We combined multiple survey years; however, we did not find any changes in the consumption of the selected food/beverage categories over these time periods. The categorization of food and beverage groups was based on the WWEIA food codes; however, the frequency of consumption of these different categories may be due to the number and types of foods/beverages within each category and could impact the results. However, we have provided information on how each food/beverage was categorized and a more detailed breakdown of the percentage of children consuming different food/beverages in the Supplemental Tables S1 and S2, respectively. Additionally, several of our estimates were not presented because the relative standard error was 40% or higher, even when multiple survey years were combined.

Lastly, this study was limited in its method of dietary intake assessment. Self-reported dietary intake assessment instruments have known systematic errors or bias; no attempt was made to address this bias [29]. Individuals may have over- or under-estimated their consumption [29,30]. Additionally, proxy respondents (even parents) of a young child may not know all of the food and beverage items a child has consumed if they were not with the child for the entire time period covered by the dietary recall.

This study also has several strengths. It is a nationally representative study with the most recently available dietary intake data, which allows us to address the nine-year data gap on food consumption patterns among young children. We were able to provide national estimates by age and race/Hispanic origin for children less than two years of age. Lastly, NHANES dietary data are collected using the AMPM which includes a multiple pass format interview, standardized probes, and memory cues to help respondents remember and describe food and beverage consumption [14]. Taking the limitations and generalizability into account may be important if these data are used to inform baseline assessments related to the upcoming dietary guidelines for children birth to 24 months of age.

## 5. Conclusions

We found that food consumption patterns in children ≤23 months of age differed by age and race/Hispanic origin. Overall, about four in ten children aged from 0 to 5 months old consumed breast milk on a given day. By race/Hispanic origin, fewer non-Hispanic black children consumed breast milk compared with their non-Hispanic white and Hispanic counterparts. Compared with children in the second year of life (12 to 23 months), a higher percentage of children at six to 11 months of age consumed vegetables and a lower percentage consumed 100% juice, sugar-sweetened beverages, snacks, or sweets. A lower percentage of non-Hispanic white children 12 to 23 months consumed sugar-sweetened beverages than Hispanic and non-Hispanic black children. Our data provide an overview of food consumption patterns of U.S. children birth to ≤23 months and may provide baseline data that is relevant for the upcoming 2020–2025 federal dietary guidelines. These data may also provide insights into potential areas that the upcoming guideline may focus on, such as fruits, vegetables, and sugar-sweetened beverage consumption.

## Figures and Tables

**Table 1:** Demographic characteristics of U.S. children birth to 23 months of age ^1^, NHANES 2009–2014.

	% (95% Confidence Interval)				
	Total Population	0 to 5 Months	6 to 11 Months	12 to 18 Months	19 to 23 Months
**Total**	-	23.7 (21.2, 26.4)	27.2 (24, 30.7)	30.4 (27.4, 33.6)	18.7 (16.4, 21.2)
	1824	512	584	445	283
**Sex**					
Male	51.7 (48.9, 54.6)	51.4 (45.8, 57.0)	51.1 (47.1, 55.2)	52.8 (47.2, 58.4)	51.3 (44.5, 58.0)
Female	48.3 (45.4, 51.1)	48.6 (43.0, 54.2)	48.9 (44.8, 52.9)	47.2 (41.6, 52.8)	48.7 (42.0, 55.5)
**Race/Hispanic origin ^2^**					
Non-Hispanic white	50.7 (44.1, 57.3)	51.8 (44.1, 59.4)	52.8 (44.0, 61.4)	51.2 (43.1, 59.2)	45.6 (36.3, 55.3)
Non-Hispanic black	13.2 (10.6, 16.3)	14.6 (11.4, 18.6)	13.6 (10.3, 17.8)	11.7 (8.6, 15.8)	13.1 (9.4, 17.9)
Hispanic	27.5 (21.9, 34)	23.1 (18.2, 28.8)	27.0 (20.3, 35.0)	29.5 (22.4, 37.8)	30.7 (22.1, 40.7)

^1^ Age in months at time of exam in Medical Examination Center (MEC). ^2^ Race/Hispanic origin subanalyses are limited to those individuals who report being either non-Hispanic white, non-Hispanic black, or Hispanic.

**Table 2:** Percentage of U.S. children birth to 23 months of age ^1^ consuming any beverages or other liquids and different food categories ^2^ by age, NHANES 2009–2014.

	% (95% Confidence Interval)			
	0 to 5 Months (*n* = 512)	6 to 11 Months (*n* = 584)	12 to 18 Months (*n* = 445)	19 to 23 Months (*n* = 283)
**Beverages or other liquids**				
Breast milk ^†, ‡^	42.9 (37.0, 49.1)	25.8 (20.2, 32.4)	7.6 (4.8, 11.8)	5.5 (2.6, 11.1)
Formula ^†, ‡^	70.5 (64.9, 75.6)	79.3 (73.6, 84.0)	9.4 (6.2, 13.9)	5.9 (2.9, 11.4)
Water ^†, ‡^	11.4 (8.4, 15.3)	48.7 (44.4, 53.0)	70.4 (64.0, 76.1)	76.6 (69.8, 82.2)
Whole milk ^†, ¥^	0	5.1 (3.3, 7.8)	70.0 (63.9, 75.4)	55.3 (47.3, 63.0)
Reduced or low or nonfat milk ^†, ¥^	0	3.1 (1.7, 5.7)	17.3 (13.1, 22.6)	30.8 (23.5, 39.2)
Flavored milk and/or milk substitute ^†, ‡^	0	*	8.8 (6.1, 12.5)	12.7 (7.1, 21.6)
100% juice ^†, ‡^	2.1 (1.2, 4)	12.3 (9.2, 16.2)	49.1 (42.0, 56.3)	51.1 (44.4, 57.8)
Sugar-sweetened beverages ^†, ‡^	1.7 (1, 2.9)	6.6 (4.7, 9.2)	31.8 (26.5, 37.6)	38.3 (29.9, 47.4)
**Food categories**				
Fruit	8.2 (5.8, 11.5)	69.0 (62.5, 74.9)	78.6 (72.2, 83.8)	68.8 (61.4, 75.3)
Vegetables, excluding white potatoes ^†, ‡^	8.1 (6.2, 10.5)	57.4 (52.0, 62.6)	48.2 (42.4, 54.1)	45.1 (36.6, 54.0)
Protein ^† ,‡^	0.8 (0.5, 1.4)	47.6 (43.1, 52.0)	87.0 (82.5, 90.4)	90.2 (84.9, 93.8)
Grains ^†, ‡^	21.1 (17.1, 25.8)	78.4 (73.5, 82.6)	85.3 (79.9, 89.4)	87.1 (81.7, 91.0)
Mixed dishes ^†, ‡^	*	25.6 (21.2, 30.5)	64.9 (59.1, 70.3)	70.8 (62.6, 77.8)
Snacks ^†, ‡^	*	12.5 (9.7, 16.1)	57.6 (51.2, 63.8)	56.7 (47.4, 65.5)
Sweets ^†, ‡^	*	13.6 (10.6, 17.1)	58.9 (51.8, 65.7)	62.9 (54.9, 70.3)

^1^ Age in months at time of exam in Medical Examination Center (MEC). ^2^ Foods and beverages contained in each category: Breast milk (human milk), Formula (ready to feed, prepared and prepared from concentrate), Whole milk (whole milk), Reduced or low or nonfat milk (reduced, low, or nonfat milk), Water (tap water and plain water), 100% juice (citrus, apple, or other fruit juice and vegetable juice), Sugar-sweetened beverages (soft drinks, fruit drinks, sports and energy drinks, nutritional beverages, smoothies and grain drinks, coffee and tea, and flavored or enhanced water), Fruit (apples, bananas, grapes, peaches and nectarines, berries, citrus fruits, melons, dried fruits, other fruits and fruit salad, baby food fruit), Vegetables excluding white potatoes (tomatoes, carrots, other red and orange vegetables, dark green leafy vegetables, lettuce and lettuce salads, string beans, onions, corn, other starchy vegetables, mixed vegetables, vegetable mixed dishes, baby food vegetables), Protein (meats, poultry, seafood, eggs and omelets, cured meats/poultry, beans, peas, legumes, nuts, seeds, processed soy products, baby food meat and dinners, baby food yogurt, cheese, yogurt), Grains (baby food cereals, ready to eat cereals, cooked cereals, breads, rolls, and tortillas, quick breads and bread products, cooked grains), Mixed dishes (mixed dishes—meat, poultry, seafood, mixed dishes—grain-based, mixed dishes—Asian, mixed dishes—Mexican, mixed dishes—Pizza, mixed dishes—sandwiches, mixed dishes—soups), Snacks (savory snacks, crackers, snack/meal bars), Sweets (sweet bakery products, candy, other desserts). * Estimate is not presented because the relative standard error (RSE) ≥40%. A zero value indicates no consumption of food group. Orthogonal linear tests for trend by age among 6–23 month olds only (*p* < 0.05); ^†^ indicates significant linear trend for age groups 6–11, 12–18, and 19–23 months; ^‡^ indicates 6–11 month olds are significantly different from 12–18 and 19–23 month olds; ^¥^ indicates 12–18 month olds are significantly different from 19–23 month olds.

**Table 3:** Percentage of U.S. children birth to 23 months of age ^1^ consuming any beverages or other liquids and different food categories ^2^ by age and race/Hispanic origin ^3^, NHANES 2009–2014.

	**0 to 5 Month-Olds**			**6 to 11 Month-Olds**		
	**Non-Hispanic White (*n* = 171)**	**Non-Hispanic Black (*n* = 104)**	**Hispanic (*n* = 178)**	**Non-Hispanic White (*n* = 189)**	**Non-Hispanic Black (*n* = 109)**	**Hispanic (*n* = 236)**
	**% (95% Confidence Interval)**			**% (95% Confidence Interval)**		
**Beverages or other liquids**						
Breast milk	49.0 (39.0, 59.1)	21.2 (13.2, 32.2) ^†^	41.9 (32.1, 52.3) ^‡^	34.8 (25.8, 44.9)	8.4 (4.1, 16.6) ^†^	19.1 (12.8, 27.6) ^†,‡^
Formula	64.0 (55.6, 71.7)	90.6 (81.7, 95.4) ^†^	74.7 (64.4, 82.8) ^†,‡^	71.5 (62.0, 79.4)	91.5 (82.7, 96.0) ^†^	86.9 (80.4, 91.5) ^†^
Water	10.6 (6.0, 18.1)	9.5 (5.4, 16.1)	13.1 (8.8, 19)	51.0 (43.6, 58.3)	33.3 (22.0, 46.9) ^†^	50.5 (44.3, 56.7) ^‡^
Whole milk	0	0	0	4.6 (2.2, 9.5)	*	7.2 (3.7, 13.3)
Reduced or low or nonfat milk	0	0	0	*	*	3.0 (1.5, 5.9)
Flavored milk and/or milk substitute	0	0	0	*	*	*
100% juice	*	*	*	8.6 (5.2, 13.9)	14.1 (8.0, 23.7)	18.2 (11.7, 27.1) ^†^
Sugar-sweetened beverages	0	2.8 (1.4, 5.6)	5.3 (2.7, 10.0)	5.5 (3.0, 9.8)	8.7 (4.7, 15.5)	8.8 (5.9, 13.0)
**Food categories**						
Fruit	6.1 (3.3, 11.0)	8.2 (4.6, 14.2)	10.7 (6.2, 17.9)	75.4 (66.2, 82.7)	49.4 (37.5, 61.4) ^†^	64.0 (55.9, 71.3) ^†,‡^
Vegetables, excluding white potatoes	7.3 (5.0, 10.6)	8.1 (3.9, 16.3)	9.1 (4.9, 16.3)	64.6 (57.1, 71.3)	52.4 (38.6, 65.7)	44.4 (35.5, 53.6) ^†^
Protein	*	*	*	49.6 (40.3, 58.9)	45.9 (35.3, 57.0)	45.5 (38.5, 52.7)
Grains	20.8 (15.0, 28.3)	34.2 (26.0, 43.4) ^†^	16.7 (12.0, 22.7) ^‡^	78.3 (69.4, 85.1)	87.5 (80.8, 92.1) ^†^	73.5 (67.0, 79.2) ^‡^
Mixed dishes	0	0	*	20.2 (14.6, 27.2)	17.2 (11.4, 25.0)	41.4 (33.7, 49.4) ^†,‡^
Snacks	0	0	0	10.4 (6.4, 16.4)	14.3 (8.1, 23.9)	15.6 (10.5, 22.4)
Sweets	*	0	*	9.2 (5.3, 15.7)	15.7 (9.7, 24.4)	20.3 (14.4, 28.0) ^†^
	**12 to 18 Month Olds**			**19 to 23 Month Olds**		
	**Non-Hispanic White (*n* = 129)**	**Non-Hispanic Black (*n* = 87)**	**Hispanic (*n* = 182)**	**Non-Hispanic White (*n* = 78)**	**Non-Hispanic Black (*n* = 57)**	**Hispanic (*n* = 112)**
	**% (95% Confidence Interval)**			**% (95% Confidence Interval)**		
**Beverages or other liquids**						
Breast milk	8.3 (4.0, 16.6)	*	6.9 (3.7, 12.4)	*	*	*
Formula	7.8 (3.9, 15.0)	8.2 (4.2, 15.4)	11.7 (6.6, 20.0)	*	*	*
Water	69.6 (58.6, 78.8)	60.7 (49.7, 70.7)	73.2 (63.9, 80.8) ^‡^	76.1 (65.1, 84.4)	75.4 (59.6, 86.4)	77.9 (63.5, 87.7)
Whole milk	73.9 (64.4, 81.6)	52.9 (39.7, 65.8) ^†^	71.1 (61.6, 79.0) ^‡^	60.2 (43.1, 75.0)	44.0 (28.5, 60.9)	52.1 (41.0, 62.9)
Reduced or low or nonfat milk	16.5 (10.5, 25.0)	23.9 (15.8, 34.3)	16.0 (10.2, 24.1) ^‡^	25.2 (13.4, 42.3)	28.3 (17.6, 42.1)	39.2 (29.2, 50.2) ^†,‡^
Flavored milk and/or milk substitute	12.9 (8.0, 20.0)	*	*	20.1 (9.2, 38.5)	*	*
100% juice	42.1 (31.1, 53.9)	63.9 (49.4, 76.3) ^†^	54.5 (46.4, 62.5) ^†,‡^	46.0 (34.8, 57.7)	66.9 (48.2, 81.5) ^†^	53.6 (43.1, 63.8) ^‡^
Sugar-sweetened beverages	26.8 (18.7, 36.9)	40.1 (30.9, 50.1) ^†^	38.9 (31.2, 47.1) ^†^	26.6 (15.4, 42.0)	45.1 (32.0, 58.8) ^†^	51.8 (36.5, 66.7) ^†^
**Food categories**						
Fruit	86.8 (76.4, 93.1)	68.2 (53.2, 80.2) ^†^	68.3 (59.0, 76.3) ^†^	72.4 (59.6, 82.4)	62.5 (46.4, 76.3)	65.8 (57.0, 73.6)
Vegetables, excluding white potatoes	57.1 (47.2, 66.5)	41.2 (30.9, 52.3) ^†^	36.8 (28.6, 45.8) ^†^	53.0 (38.4, 67.1)	39.4 (23.1, 58.4)	37.2 (24.6, 51.8) ^†^
Protein	90.9 (82.8, 95.4)	83.1 (73.7, 89.6)	86.2 (79.8, 90.7)	92.8 (82.3, 97.3)	90.1 (82.4, 94.7)	84.6 (77.2, 89.9) ^†^
Grains	86.8 (77.1, 92.8)	89.7 (78.3, 95.4)	77.8 (70.8, 83.4) ^†,‡^	91.2 (83.0, 95.6)	89.2 (73.5, 96.1)	78.1 (65.6, 87.0) ^†,‡^
Mixed dishes	61.0 (50.7, 70.3)	74.0 (63.5, 82.3) ^†^	68.4 (60.9, 75.0) ^†^	63.7 (49.1, 76.1)	76.0 (59.4, 87.3)	77.1 (65.6, 85.6) ^†^
Snacks	67.4 (57.4, 76.1)	60.1 (47.8, 71.3)	45.6 (36.3, 55.3) ^†,‡^	61.7 (43.8, 76.8)	70.4 (53.7, 83.0)	47.0 (36.5, 57.8) ^†,‡^
Sweets	58.8 (47.3, 69.5)	62.8 (51.5, 73.0)	58.3 (48.4, 67.6)	60.3 (45.3, 73.5)	62.1 (49.6, 73.2)	64.1 (53.6, 73.4)

^1^ Age in months at time of exam in Medical Examination Center (MEC). ^2^ Foods and beverages contained in each category: Breast milk (human milk), Formula (ready to feed, prepared and prepared from concentrate), Whole milk (whole milk), Reduced or low or nonfat milk (reduced, low, or nonfat milk), Water (tap water and plain water), 100% juice (citrus, apple, or other fruit juice and vegetable juice), Sugar-sweetened beverages (soft drinks, fruit drinks, sports and energy drinks, nutritional beverages, smoothies and grain drinks, coffee and tea, and flavored or enhanced water), Fruit (apples, bananas, grapes, peaches and nectarines, berries, citrus fruits, melons, dried fruits, other fruits and fruit salad, baby food fruit), Vegetables excluding white potatoes (tomatoes, carrots, other red and orange vegetables, dark green leafy vegetables, lettuce and lettuce salads, string beans, onions, corn, other starchy vegetables, mixed vegetables, vegetable mixed dishes, baby food vegetables), Protein (meats, poultry, seafood, eggs and omelets, cured meats/poultry, beans, peas, legumes, nuts, seeds, processed soy products, baby food meat and dinners, baby food yogurt, cheese, yogurt), Grains (baby food cereals, ready to eat cereals, cooked cereals, breads, rolls, and tortillas, quick breads and bread products, cooked grains), Mixed dishes (mixed dishes—meat, poultry, seafood, mixed dishes—grain-based, mixed dishes—Asian, mixed dishes—Mexican, mixed dishes—Pizza, mixed dishes—sandwiches, mixed dishes—soups), Snacks (savory snacks, crackers, snack/meal bars), Sweets (sweet bakery products, candy, other desserts). ^3^ Race/Hispanic origin subanalyses are limited to those individuals who report being either non-Hispanic white, non-Hispanic black, or Hispanic. All pair-wise comparisons were conducted within age groups (*t*-test, *p* < 0.05); ^†^ indicates significantly different from non-Hispanic whites; ^‡^ indicates significantly different from non-Hispanic blacks within each age group. * Estimate is not presented because the relative standard error (RSE) ≥ 40%. A zero value indicates no consumption of food group.

## Supplementary Materials

The following are available online at http://www.mdpi.com/2072-6643/9/9/942/s1, Table S1: Food and beverage categories and associated subcategories and the United States Department of Agriculture’s (USDA) What We Eat in America food categorization codes, Table S2: Percentage of U.S. children birth to 23 months of age consuming any fruit, vegetables, protein, grains, mixed dishes, snacks, sweets, or baby food, NHANES 2009–2014.

## Abbreviations

AMPM	Automated Multiple Pass Method
CI	Confidence Intervals
FITS	Feeding Infants and Toddlers Study
MEC	Mobile Examination Center
NHANES	National Health and Nutrition Examination Survey
NIS	National Immunization Survey
USDA	United States Department of Agriculture
WIC	Special Supplemental Nutrition Program for Women, Infants, and Children
WWEIA	What We Eat in America
