# Supplementary material for: Food Consumption Patterns among U.S. Children from Birth to 23 Months of Age, 2009–2014
Source: Nutrients. 2017 Aug 26;9(9):942. doi: 10.3390/nu9090942 (PMC5622702; doi:10.3390/nu9090942)
Supplement: Supplementary file 1 [file nutrients-09-00942-s001.zip › Supplemental Material_TableS1.docx]

Supplemental Material

**Table S1.** Food and beverage categories and associated subcategories and the United States Department of Agriculture’s (USDA) What We Eat in America food categorization codes.

| **Categories** | **Subcategory** | **USDA What We Eat in America Food Codes** |
| --- | --- | --- |
| **Beverages or other liquids** |  | |
| Breast milk |  | 9602 |
| Formula | Ready to feed, prepared, and prepared from concentrate | 9402, 9404, 9406 |
| Whole milk | Whole milk | 1002 |
| Reduced or low or nonfat milk | Reduced, low fat, or nonfat milk | 1004, 1006, 1008 |
| Flavored milk and/or milk substitute | Flavored milk (whole, reduced fat, low fat, or nonfat) | 1202, 1204, 1206, 1208 |
|  | Milk substitutes, milk shakes and other dairy drinks | 1404, 1402 |
| Water | Tap water, plain water | 7702, 7704 |
| 100% juice | Citrus juice, apple juice, other fruit juice, vegetable juice | 7002, 7004, 7006, 7008 |
| Sugar-sweetened beverages | Sweetened beverages (soft drinks, fruit drinks, sports and energy drinks, nutritional beverages, smoothies and grain drinks) | 7202, 7204, 7206, 7208, 7220 |
|  | Coffee and tea | 7302, 7304 |
|  | Flavored or enhanced water | 7802, 7804 |
| **Food categories** |  | |
| Fruit | Apples | 6002 |
|  | Bananas | 6004 |
|  | Grapes | 6006 |
|  | Peaches and nectarines | 6008 |
|  | Berries | 6010 |
|  | Citrus fruits | 6012 |
|  | Melons | 6014 |
|  | Dried fruits | 6016 |
|  | Other fruits and fruit salads | 6018 |
|  | Baby food: fruit | 9004 |
| Vegetables, excluding white potatoes | Tomatoes, carrots, other red and orange vegetables | 6402, 6404, 6406 |
|  | Dark green leafy vegetables, lettuce and lettuce salads, string beans | 6408, 6410, 6412 |
|  | Other vegetables (onion, corn, other starchy vegetables, mixed vegetables, vegetable mixed dishes) | 6414, 6416, 6418, 6420, 6422 |
|  | Baby food: vegetables | 9006 |
| Protein | Meats | 2002, 2004, 2006, 2008, 2010 |
|  | Poultry | 2202, 2204, 2206 |
|  | Seafood | 2402, 2404 |
|  | Eggs and omelets | 2502 |
|  | Cured meats/poultry | 2602, 2604, 2606, 2608 |
|  | Beans, peas, legumes, nuts, seeds, and processed soy products | 2802, 2804, 2806 |
|  | Baby food: meat and dinners | 9008 |
|  | Baby food: yogurt | 9010 |
|  | Cheese and yogurt | 1602, 1604, 1802, 1804, 1820, 1822 |
| Grains | Baby food: cereals | 9002 |
|  | Ready to eat cereals | 4602, 4604 |
|  | Cooked cereal | 4802, 4804 |
|  | Breads, rolls, tortillas | 4202, 4204, 4206, 4208 |
|  | Quick breads and bread products | 4402, 4404 |
|  | Cooked grains | 4002, 4004 |
| Mixed dishes | Mixed dishes—Meat, poultry, seafood | 3002, 3004, 3006 |
|  | Mixed dishes—Grain-based | 3202, 3204, 3206, 3208, |
|  | Mixed dishes—Asian | 3402, 3404, 3406 |
|  | Mixed dishes—Mexican | 3502, 3504, 3506 |
|  | Mixed dishes—Pizza | 3602 |
|  | Mixed dishes—Sandwiches | 3702, 3703, 3704, 3706, 3708 |
|  | Mixed dishes—Soup | 3802 |
| Snacks | Savory snacks | 5002, 5004, 5006, 5008 |
|  | Crackers | 5202, 5204 |
|  | Snack/meal bars | 5402, 5404 |
| Sweets | Sweet bakery products | 5502, 5504, 5506 |
|  | Candy | 5702, 5704 |
|  | Other desserts | 5802, 5804, 5806 |
| **Additional food categories (Presented in Supplemental Table S2)** | |  |
| Vegetables: white potatoes | White potatoes (baked or boiled, French fries and other fried white potatoes, mashed and white potato mixtures) | 6802, 6804, 6806 |
| Baby foods | Baby food: Snacks and sweets | 9012 |
|  | Baby food: Juice | 9202 |
|  | Baby food: Water | 9204 |
